# Evidence for ligninolytic activity of the ascomycete fungus *Podospora anserina*

**DOI:** 10.1186/s13068-020-01713-z

**Published:** 2020-04-16

**Authors:** Gijs van Erven, Anne F. Kleijn, Aleksandrina Patyshakuliyeva, Marcos Di Falco, Adrian Tsang, Ronald P. de Vries, Willem J. H. van Berkel, Mirjam A. Kabel

**Affiliations:** 1 Laboratory of Food Chemistry, Wageningen University and Research, Bornse Weilanden 9, 6708 WG Wageningen, The Netherlands; 2 Fungal Physiology, Westerdijk Fungal Biodiversity Institute and Fungal Molecular Physiology, Utrecht University, Uppsalalaan 8, 3584 CT Utrecht, The Netherlands; 3 Centre for Structural and Functional Genomics, Concordia University, 7141 Sherbrooke Street West, Montréal, Québec H4B 1R6 Canada

**Keywords:** Biomass, Enzymes, Lignin, Laccase, py-GC–MS, NMR spectroscopy, Proteomics, Secretomics

## Abstract

**Background:**

The ascomycete fungus *Podospora anserina* has been appreciated for its targeted carbohydrate-active enzymatic arsenal. As a late colonizer of herbivorous dung, the fungus acts specifically on the more recalcitrant fraction of lignocellulose and this lignin-rich biotope might have resulted in the evolution of ligninolytic activities. However, the lignin-degrading abilities of the fungus have not been demonstrated by chemical analyses at the molecular level and are, thus far, solely based on genome and secretome predictions. To evaluate whether *P. anserina* might provide a novel source of lignin-active enzymes to tap into for potential biotechnological applications, we comprehensively mapped wheat straw lignin during fungal growth and characterized the fungal secretome.

**Results:**

Quantitative ^13^C lignin internal standard py-GC–MS analysis showed substantial lignin removal during the 7 days of fungal growth (24% w/w), though carbohydrates were preferably targeted (58% w/w removal). Structural characterization of residual lignin by using py-GC–MS and HSQC NMR analyses demonstrated that C_α_-oxidized substructures significantly increased through fungal action, while intact β-*O*-4′ aryl ether linkages, *p*-coumarate and ferulate moieties decreased, albeit to lesser extents than observed for the action of basidiomycetes. Proteomic analysis indicated that the presence of lignin induced considerable changes in the secretome of *P. anserina*. This was particularly reflected in a strong reduction of cellulases and galactomannanases, while H_2_O_2_-producing enzymes clearly increased. The latter enzymes, together with laccases, were likely involved in the observed ligninolysis.

**Conclusions:**

For the first time, we provide unambiguous evidence for the ligninolytic activity of the ascomycete fungus *P. anserina* and expand the view on its enzymatic repertoire beyond carbohydrate degradation. Our results can be of significance for the development of biological lignin conversion technologies by contributing to the quest for novel lignin-active enzymes and organisms.

## Background

With the increasing interest in biological lignocellulose valorization strategies, there is a quest for novel lignocellulose-acting enzymes and organisms producing such enzymes [1]. The ascomycete fungus *Podospora anserina* has long been studied for its lifecycle characteristics, particularly regarding its short growth phase, but more recently also has sparked biotechnological interest [2–6]. The fungus produces a wide variety of carbohydrate-active enzymes, including several cellulases, xylanases and lytic polysaccharide monooxygenases (LPMOs) and promises to be an efficient microbial enzyme factory because of its ease of genetic manipulation and fast growth [5–8]. Indeed, *P. anserina* enzymes were shown to be of biotechnological potential, being able to enhance the saccharification efficiencies of enzymes commonly used in industry [9, 10].

As a late colonizer of herbivorous dung, *P. anserina* is expected to specifically degrade the more recalcitrant fraction of lignocellulose [11]. Furthermore, the fungus should be able to detoxify metabolites remaining in the dung and those resulting from the deconstruction of the macromolecules it contains [6]. The latter detoxifying ability presumably strongly depends on cytochrome P450 enzymes, of which many are present in the genome of *P. anserina* [5]. The fungus, furthermore, showed a dependency on catalases to grow on lignocellulose, which was related to the regulation of H_2_O_2_ levels [12]. Considering the biotope, the fungus might besides its well-studied carbohydrate-degrading enzymatic arsenal also possess ligninolytic activity, which could broaden the scope of the exploitation of *P. anserina* in biomass upgrading approaches [13]. The suggestion of possible ligninolytic activity is based on the ability of the fungus to grow on a variety of lignocellulosic substrates, including nearly pure lignin (heavily pretreated, Kraft lignin), and its genome that encodes several putative lignin-active enzymes including laccases and H_2_O_2_-producing oxidoreductases [5, 12, 14]. Actually, the genome of *P. anserina* is, amongst ascomycetes, characterized by the highest number of genes encoding auxiliary activities (AA), encompassing oxidoreductases only, as accommodated in the carbohydrate-active enzymes database (CAZy; www.cazy.org) [15]. Secretome analyses revealed that some of these enzymes were produced and secreted during growth on lignocellulose [10, 16]. Furthermore, deletion of some of the laccase-encoding genes reduced the ability of the fungus to grow on wood shavings, suggesting that they are indeed involved in wood degradation [17]. However, the actual degradation of lignin has not been shown by chemical analyses of the substrates; and as such, the ligninolytic capability of *P. anserina* remains to be further elucidated.

Recently, we have demonstrated that quantitative pyrolysis-GC–MS analysis is exceptionally useful for mapping the extent of fungal plant biomass delignification as well as for unravelling the underlying ligninolysis mechanisms [18–20]. Importantly, this technique can highlight minor structural changes in the remaining lignin after short periods of fungal growth, an unconditional requirement when studying the structural changes induced by micro-organisms with a short growth cycle [19, 20].

In this work, the ligninolytic activity of *P. anserina* was unambiguously confirmed through comprehensive substrate analysis after growth of the fungus on wheat straw lignin. Quantitative py-GC–MS analyses revealed substantial lignin removal and an increase in C_α_-oxidized moieties indicative of oxidative degradation. HSQC NMR analysis further substantiated these findings by showing a decrease of intact β-*O*-4′ aryl ether linkages concomitant with the increase of C_α_-oxidized substructures. In addition, *p*-coumarate and ferulate moieties were decreased, which suggests feruloyl esterase activity during fungal growth. Secretome analysis indicated that ligninolysis likely involved laccases and H_2_O_2_-producing enzymes.

## Results and discussion

### Cultivation setup

To simplify the detection of early markers of ligninolytic activity of *P. anserina*, we used cultivation conditions that would allow us to evaluate changes in both lignin structure and in the fungal secretome [21]. To this end, we incubated the fungus on pre-isolated lignin from wheat straw that was combined with the hemicellulose fraction of the straw, i.e., glucuronoarabinoxylan (GAX), instead of growing the fungus on unfractionated wheat straw. This enabled us to characterize the residual substrates directly by detailed HSQC NMR analysis, which would otherwise require lignin isolation to reach the same level of structural insight [18]. In addition, through this setup the secretome of *P. anserina* could be compared to growth on GAX alone. The isolated lignin was structurally representative of wheat straw lignin and of high purity (90% w/w) (Additional file 1: Figure S1, Table S1 and S2) [22]. The hemicellulose that was used in the fermentations introduced negligible amounts of lignin and only slightly contributed to the total ferulate content as inferred from the 4-vinylguaiacol product in the pyrogram (Additional file 1: Figure S1). HSQC NMR analysis of the GAX failed to show any ferulate-specific correlation peaks, presumably due to poor solubilization/gelling of the sample. A total esterified content of (di) ferulates in the GAX sample was estimated at 2.9% (w/w) by our research group in earlier research [23].

### Fate of carbohydrates and lignin after growth of *P. anserina*

**Fig. 1:**
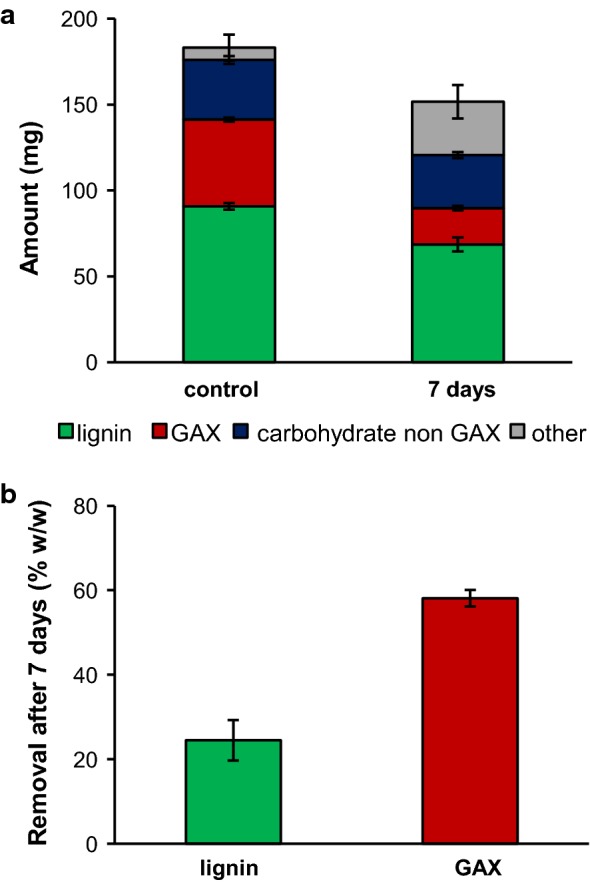
Absolute recoveries (**a**) and removal (**b**) of lignin and glucuronoarabinoxylan (GAX) after 7 days of growth of *P. anserina* on wheat straw lignin isolate and insoluble wheat glucoronoarabinoxylan. Total insoluble and water-soluble fractions based on compositional analysis by using quantitative ^13^C-IS py-GC–MS (lignin) and constituent monosaccharide analysis after H_2_SO_4_ hydrolysis (carbohydrates). Others represent residual dry matter. Note that the initial lignin isolate contained some residual carbohydrates (10% w/w) and initial hemicellulose contained some residual cellulose (16% w/w) [23]

After 7 days of growth of *P. anserina*, substantial amounts of mycelium could be observed by visual inspection. Mass balance analysis revealed that the fungus had degraded and removed considerable amounts of the initial substrate (Fig. 1a). As fungal mycelium is generally composed of carbohydrates which do not contain the glucuronosyl, arabinosyl and xylosyl moieties of the hemicellulosic substrate, we analyzed the recoveries of GAX and other carbohydrates separately [24]. *P. anserina* was able to degrade and remove substantial amounts of lignin (24% w/w) besides GAX (58% w/w) during growth (Fig. 1b).

As expected from its wealth of carbohydrate-active enzymes [5], the fungus degraded the hemicellulosic carbohydrates with a clear preference. Still, though ligninolytic activity of *P. anserina* has previously been considered [13], this is the first evidence that the fungus is actually able to degrade and remove the aromatic biopolymer.

The ligninolytic capacity of *P. anserina*, i.e., the extent of lignin removal during 7 days of fungal growth, was remarkably high for an ascomycete fungus, in comparison to previous studies that employed radiolabeled lignin preparations [25, 26]. However, it should be taken into account that we grew the fungus on pre-isolated lignin under submerged fermentation conditions, in which lignin was presumably more accessible compared to whole cell wall preparations and solid-state incubations. Furthermore, whether *P. anserina* completely mineralized lignin to CO_2_ or depolymerized it to such an extent that the resulting volatile degradation products were removed during freeze-drying remains to be further investigated.

### Structural characterization of lignin after growth of *P. anserina*

The structural features of the residual lignin further substantiated the ligninolytic action of *P. anserina* (Table 1). A slight, but significant, increase (10%) in C_α_-oxidized substructures suggested that oxidative degradation forms the basis of the lignin removal observed. Interestingly, diketone products, pyrolysis markers for dihydroxyphenylketones, had increased significantly after fungal growth from 0.16 ± 0.00% to 0.25 ± 0.00% of the relative abundance of lignin-derived pyrolysis products; and consequently explained a major part of the increase of the C_α_-oxidized substructures. These markers have also been observed after the action of the white-rot fungus *Ceriporiopsis subvermispora* [19] and for laccase-mediator systems [27], suggesting that *O*-4′-cleavage of β-*O*-4′ aryl ethers is likely one of the involved ligninolysis routes.

**Table 1:** Quantitative ^13^C-IS py-GC–MS structural characterization of untreated and 7 days *P. anserina*-treated wheat straw lignin; corrected for relative response factors and relative abundance of ^13^C analogues

	Control	7 days
Lignin subunits (%)		
H	8.9 ± 0.1	8.6 ± 0.0**
G	60.9 ± 0.5	59.3 ± 0.2**
S	30.2 ± 0.5	32.1 ± 0.2**
S/G	0.50 ± 0.0	0.54 ± 0.0**
Structural moieties (%)		
Unsubstituted	6.4 ± 0.3	6.6 ± 0.1
Methyl	3.0 ± 0.2	3.3 ± 0.0*
Vinyl	32.4 ± 1.1	30.4 ± 0.3*
4-VP^a^	7.2 ± 0.1	6.6 ± 0.0**
4-VG^b^	21.7 ± 1.1	19.9 ± 0.3
C_α_-ox	4.0 ± 0.1	4.3 ± 0.1*
Diketones	0.16 ± 0.0	0.25 ± 0.0**
C_β_-ox^c^	2.5 ± 0.1	2.5 ± 0.0
C_γ_-ox	48.2 ± 1.1	49.1 ± 0.4
Miscellaneous	3.6 ± 0.1	3.8 ± 0.0
PhC_γ_^d^	52.6 ± 1.1	53.9 ± 0.4
PhC_γ_-diketones^e^	52.4 ± 1.1	53.6 ± 0.4

Sum based on structural classification according to van Erven et al. [18, 19]. Average and standard deviation of analytical duplicates of biological triplicates. Structural features for the combined fractions weighted on the basis of the lignin mass balance

^a^4-Vinylphenol. ^b^4-Vinylguaiacol. ^c^Excluding diketones. ^d^Phenols with intact α, β, γ carbon side chain. ^e^Phenols with intact α, β, γ carbon side chain, excluding diketones

Significant differences calculated by two-tailed t-test (*p < 0.05, **p < 0.01)

However, at the observed extents of lignin removal (24% w/w, Fig. 1), we would have expected to see more prominent changes in the structural characteristics of the residual lignin. For several white-rot fungal-treated samples we, and others, have previously observed an approximate doubling of total C_α_-oxidized moieties at comparable extents of delignification [18, 28, 29], and *C. subvermispora* showed a 16-fold increase in diketone markers [20]. These observations would suggest that different (or additional) degradation routes underlie delignification, as elaborated below. Besides increased C_α_-oxidized substructures, the action of *P. anserina* led to a substantial decrease in vinyl products (Table 1).

These products are largely derived from the decarboxylation of hydroxycinnamates upon pyrolysis [30, 31]. In particular, the decrease of 4-vinylphenol, primarily formed from *p*-coumarate, suggests that these moieties were targeted, at least to some extent, during fungal growth. Likewise, the decrease in 4-vinylguaiacol suggests the removal of ferulic acid moieties.

**Fig. 2:**
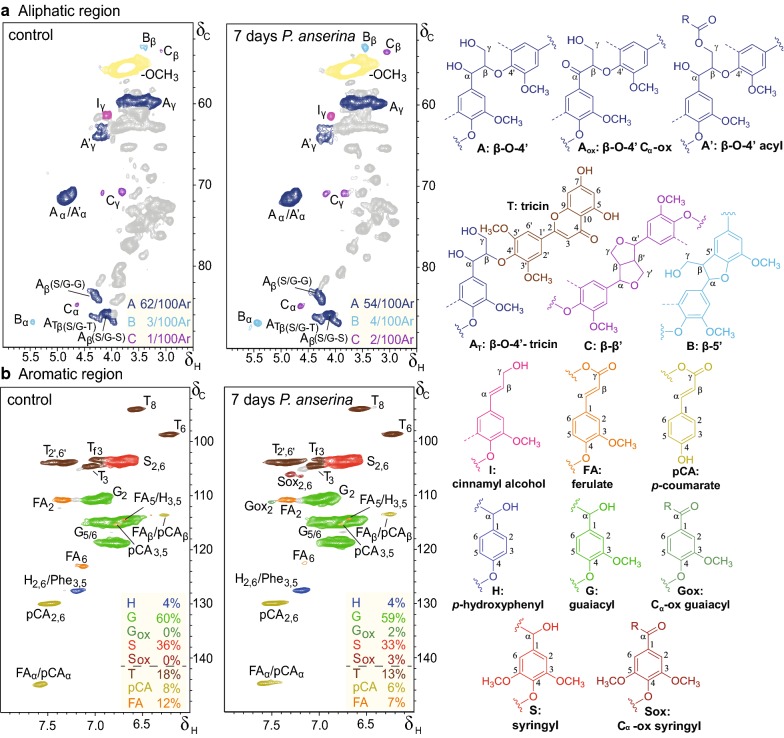
Aliphatic (**a**) and aromatic (**b**) regions of ^1^H-^13^C HSQC NMR spectra of untreated and 7 days *P. anserina*-treated wheat straw lignin water-insoluble residues. Unassigned signals are shown in gray. Colored boxes show semiquantitative analysis of the volume integrals, with interunit linkages per 100 subunits (aromatic rings, Ar), relative distribution of subunits (%) and T, *p*CA and FA relative to total subunits (%). Dotted lines represent –H or –OCH_3_. Wavy lines indicate main positions for further coupling. Unassigned signals are shown in gray

To further corroborate our findings, we structurally characterized the lignin fractions in more detail by ^1^H–^13^C HSQC NMR analysis. Indeed, clear structural changes could be observed in the spectra and were further resolved by semiquantitative analysis of the volume integrals (Fig. 2).

In the aromatic region of the NMR spectra of the treated lignin residue samples, C_α_-oxidized substructures became evident and were shown to comprise approximately 4% of the total subunits. Concurrently with the increase of C_α_-oxidized substructures, intact β-*O*-4′ aryl ether interunit linkages decreased from 62 to 54 linkages/100 subunits, implying that the oxidized substructures resulted from interunit bond-cleavage (Fig. 2). In contrast to the β-*O*-4′ aryl ether substructures, the more condensed phenylcoumaran (β-5′) and resinol (β-β’) structural elements appeared to be more resistant against degradation by *P. anserina* and as a result they relatively accumulated in the fungal-grown residues, albeit slightly (Fig. 2, Table 2).

**Table 2:** Semiquantitative ^1^H-^13^C HSQC NMR structural characterization of untreated and 7 days *P. anserina*-treated wheat straw lignin

	Control	7 days
Lignin subunits (%)^a^		
H	4	4
G	61	59
G_ox_	0	2
S	35	34
S_ox_	0	2
S/G	0.6	0.6
Hydroxycinnamates (%)^b^		
*p*-Coumarate	8	6
Ferulate	10	8
Flavonolignin (%)^b^		
Tricin	14	15
Lignin interunit linkages (%)^b,c^		
β-*O*-4′ Aryl ethers	63 (93)	59 (92)
β-5′ Phenylcoumarans	4 (5)	4 (6)
β-β’ Resinols	1 (2)	1 (2)
Total	68 (100)	64 (100)

Structural features for the combined fractions weighted on the basis of the lignin mass balance

^a^Relative distribution of lignin subunits (H + G+G_ox_ + S+S_ox_ = 100)

^b^Relative volume integral of substructure versus volume integral of total lignin subunits

^c^Relative distribution of total interunit linkages in parentheses

In line with our observations by py-GC–MS analysis, *p*-coumarate moieties decreased during fungal growth. Because these *p*-coumarates are solely present as γ-esters, their removal implies hydroxycinnamic acid esterase activity [32]. Similar activity likely also caused the slight reduction in ferulates, in the case of ester-bound moieties to GAX, though oxidative degradation of ether-bound ferulates cannot be excluded [32]. When the structural features were weighted for the contributions of the various fractions to the total remaining lignin, the structural changes mediated by fungal action could still be discerned (Table 2).

Note that even though in HSQC NMR spectra C_β_–H_β_ correlation peaks are resolved for various substructures of β-*O*-4′ aryl ether linkages [20, 33], the hemicellulosic substrate presented an overlapping peak with the β-*O*-4′-S/G-S signal at δ_C_/δ_H_ 85.97/3.96 ppm in this experimental setup. Therefore, we quantified the C_α_–H_α_ correlation peaks instead, as previously described by del Río et al. [34].

The aforementioned structural observations by py-GC–MS and HSQC NMR analyses might be the result of a preferential degradation of phenolic subunits. These subunits generally comprise 10–30% of the lignin polymer and are more susceptible to oxidation [35, 36]. Hence, these phenolic subunits might be degraded to a greater extent than their non-phenolic counterparts. However, the oxidation of phenolic moieties might also drive (re)polymerization reactions [37].

In the absence of mediators, or so-called electron-shuttles, it can be expected that laccases, the only ‘true’ ligninolytic enzymes of *P. anserina*, are able to oxidize phenolic substructures only [38]. With the necessity of being the end-groups of the growing lignin polymer, the cleavage of these units might have proceeded in a sort of ‘peeling pattern’, leaving the remaining polymer principally intact, or at least not distinguishable from native lignin apart from a (slight) reduction of molecular weight [39]. Consequently, this could explain why we observed relatively limited structural modification of the residual lignin after partial delignification. Even though some lignin-derived phenols could have acted as mediators [40, 41], thereby broadening the oxidative capacity of laccases to also oxidize non-phenolic subunits, this would presumably have been accompanied by more severe lignin modification.

### Structural changes in light of the fungal secretome

In order to relate the observed structural changes to potential enzymatic action and evaluate whether *P. anserina* induces a dedicated ligninolytic machinery in the presence of lignin, we performed proteomic analysis of the secreted proteins during growth of the fungus on GAX and GAX + lignin. These analyses identified a total of 430 proteins, of which 201 (47%) could be assigned to CAZymes (Additional file 3). Of these secreted CAZymes, 165 enzymes were annotated to a putative plant cell wall-active function, covering approximately 50% of the 333 CAZymes encoded in the genome of *P. anserina* (*mat *+ v1.0) with a putative plant cell wall-active function (Additional file 3). Most of these enzymes were secreted in both growth conditions, while the number of unique enzymes for GAX (19 enzymes) and GAX + lignin (15 enzymes) was comparable. For both substrates, these unique plant cell wall-active enzymes summed up to 1% of the total relative abundance only. The total relative abundance of plant cell wall-active enzymes was approximately 67% and 54% for GAX and GAX + lignin, respectively (Additional file 2: Figure S2). Unique and significantly induced proteins other than plant cell wall-active CAZymes during growth of *P. anserina* on GAX and GAX + lignin are reported in Additional file 3: Table S3. Four proteins with unknown function were significantly induced in the presence of lignin and especially the 4.4-fold induction of protein #397192, as most abundant secreted protein detected (at 13.5% of the total relative abundance), deserves to be mentioned here (Additional file 3). The elucidation of the (putative) function of these lignin-induced unknown proteins might provide further insight into novel enzyme activities involved in lignin conversion in general and in the ligninolytic action of *P. anserina* in particular.

**Fig. 3:**
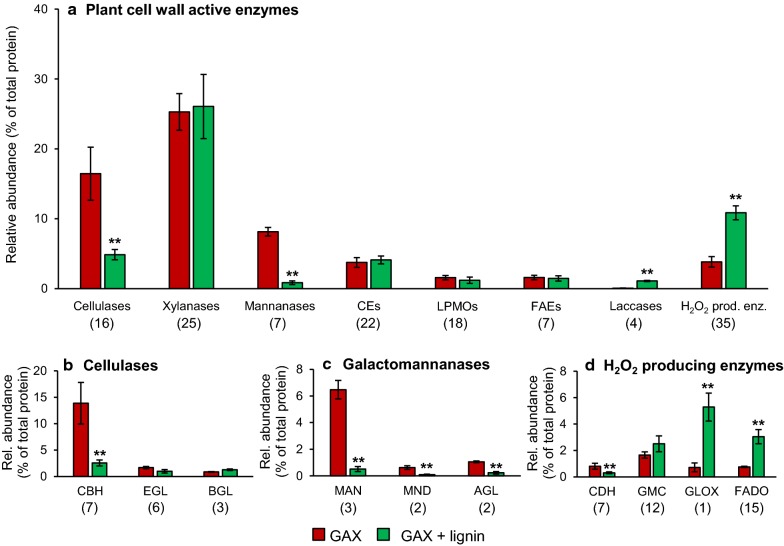
Proteomics analysis of secreted plant cell wall-active enzymes during growth of *P. anserina* on glucuronoarabinoxylan (GAX) and GAX + lignin. Enzymes were grouped on the basis of overall functionality (**a**) and further subdivided into more specific activities in (**b**), (**c**), (**d**). Number of detected enzymes per group in parentheses. Total number of encoded and secreted CAZymes in Additional file 3. CEs carbohydrate esterases; LPMOs lytic polysaccharide monooxygenases; FAEs feruloyl esterases; CBH cellobiohydrolases; EGL endoglucanases, BGL β-glucosidases; MAN endomannanases; MND β-mannosidases; AGL α-galactosidase; CDH cellobiose dehydrogenases; GMC glucose–methanol–choline oxidoreductases; GLOX glyoxal oxidases; FADO FAD-linked oxidases. Significant differences calculated by two-tailed t-test (**p < 0.01)

Irrespective of the presence of lignin, carbohydrate hydrolases made up the majority of the detected plant cell wall-active enzymes during growth (Fig. 3). However, lignin did induce a relative shift from a hydrolytic towards a more oxidative enzymatic degradation pattern. Most strikingly, cellulases (3.4-fold) and galactomannanases (9.8-fold) were strongly decreased in the presence of lignin, while laccases (20-fold) and H_2_O_2_ producing enzymes (threefold) were strongly increased (Fig. 3).

The decrease in cellulases was predominately explained by a reduction in cellobiohydrolases (5.4-fold) (Fig. 3b) and this reduction was primarily caused by two enzymes (GH7 #368104, 23-fold and GH6 #301916, 20-fold) (Additional file 2: Figure S3A). Even though endoglucanases overall did not decrease significantly (1.7-fold), one enzyme (GH5 #382814) was basically absent in the presence of lignin (Additional file 2: Figure S3B). Though the overall β-glucosidase abundance was not largely affected (1.5-fold decrease), a GH3 β-glucosidase (#166682) decreased 32-fold, while another GH3 β-glucosidase (#330692) increased 14-fold in the presence of lignin (Additional file 2: Figure S3C).

The overall decrease in galactomannanases could be attributed to endomannanases, β-mannosidases and α-galactosidases (Fig. 3c) and mostly to a 26-fold reduction in a GH5_7 endomannanase (#132963) (Additional file 2: Figure S3D–F). The latter enzyme has previously been characterized (*Pa*Man5A) and was able to enhance the degradation of spruce polysaccharides [9]. Mäkelä et al. found that the production of this enzyme was heavily dependent on the substrate used for cultivation and our findings now indicate that this dependence could be due to the presence of lignin [10].

Overall, LPMOs did not decrease with the cellulase system in the presence of lignin (Fig. 3). Interestingly, an AA16 LPMO (#3768620) was detected in the secretome, which in response to the presence of lignin decreased 22-fold (Additional file 2: Figure S3G). Furthermore, it is worth mentioning that one LPMO (AA9, #179198, characterized as PaLPMO9B) was 2.7-fold decreased while three other AA9 LPMOs, #403339 (PaLPMO9C), #307960 (PaLPMO9E) and #287378 (PaLPMO9A), increased 10.7-, 9.7- and 4.5-fold, respectively (Additional file 2: Figure S3G) [7, 42]. The increase in CBM1 harboring PaLPMOs 9A and 9E, characterized to work on cellulose rather strictly, is interesting given the severe reduction in cellulases [7]. This could indicate that presence of lignin triggered the fungus to produce LPMOs ahead of cellulases and could, consequently, indicate that the enzymes might act synergistically, but not necessarily concurrently [43, 44]. The activity of the induced LPMOs has been demonstrated to be boosted by cellobiose dehydrogenases (CDHs) and they have been considered to function together in vivo [7, 42]. However, the overall abundance of cellobiose dehydrogenases (CDHs) was 2.6-fold reduced in the presence of lignin (Fig. 3), which contends their putative concerted action with LPMOs, at least from the point of view that they do not seem to be co-regulated per se during growth of *P. anserina*. Alternatively, the secreted LPMOs might have depended on other electron transfer systems involving the other H_2_O_2_ producing enzymes that were strongly induced in the presence of lignin, as will be elaborated below [45, 46].

The total xylanase machinery was not significantly affected by the presence of lignin (Fig. 3) and the production of various endoxylanases and arabinofuranosidases allowed the fungus to efficiently degrade GAX during its growth (~ 60% w/w, Fig. 1). Even though the grouped xylanases did not show an overall significant change in the presence of lignin, clear differences could be observed at the individual enzyme level (Additional file 2: Figure S3H–I). A GH10 endoxylanase (#388128) was 46-fold reduced together with the reduction of two GH11 endoxylanases (#405228 fivefold, #428889 74-fold) in the presence of lignin, while another GH10 endoxylanase (#333275) increased 5.6-fold (Additional file 2: Figure S3H). Even though in terms of total endoxylanase abundance these effects cancel out, it is interesting to observe that the presence of lignin can trigger a shift in the production of certain endoxylanases, with potentially different functionalities and/or specificities.

Likewise, the presence of lignin induced a 2.9-fold reduction in two predicted GH43 arabinofuranosidases (#438134 and #300046), while the GH51 (#109488, PaAbf51A) and GH62 (#434801, PaAbf62A) arabinofuranosidases, respectively, increased by sixfold and 4.8-fold (Additional file 2: Figure S3I). In line with the induction by lignin, Mäkelä et al. showed that when *P. anserina* was grown on wheat straw, the latter GH62 arabinofuranosidase was produced in greater abundance than when grown on cottonseed and soybean hulls [10]. The increase of the two arabinofuranosidases (PaAbf51A and PaAbf62A) is remarkable in the sense that they have been characterized to exhibit low activity on polymeric substrates, including wheat arabinoxylan (low viscosity), and were shown to cleave terminal arabinosyl units only [9].

The overall relative abundance of carbohydrate (mostly acetyl xylan, AXE) and feruloyl esterases (FAEs) was not significantly different in the presence and absence of lignin (Fig. 3). Still, a putative CE15 4-*O*-methyl-glucuronyl methylesterase (#118359) was not produced in the presence of lignin, while a putative CE12 rhamnogalacturonan acetylesterase (#381424) was decreased by a factor 6.1 (Additional file 2: Figure S3J). Conversely, four other carbohydrate esterases significantly increased (CE1 putative acetylxylan esterase (AXE) with carbohydrate-binding module specific for cellulose (CBM1) #284003 threefold, CE3 #281974 12-fold, CE5 with CBM1 #110452 fivefold and CE5 putative cutinase #36734 24-fold). Apart from a severe reduction in a CE1 FAE #312581 (71-fold), the abundance of the other individual FAEs was not significantly different in the presence of lignin (Additional file 2: Figure S3K). These FAEs have often been suggested to be involved in disentangling lignin and hemicellulose, and therefore, their production might be induced by the presence of lignin or related substructures [10, 47]. Apparently, the hemicellulosic substrate used in the current cultivations without lignin already induced these enzymes and the presence of lignin did not further increase their production. Although not changed in relative abundance, the FAEs were still most likely responsible for the reduction in ferulate and *p*-coumarate moieties in the residual lignin after growth of *P. anserina* (Table 2) [23].

The highly significant increase in H_2_O_2_-producing enzymes (2.9-fold) was particularly due to a single putative AA5_1 glyoxal oxidase (GLOX, #15405), which increased 7.3-fold (Fig. 3d). In addition, FAD-linked oxidases (FADO) overall fourfold increased in the presence of lignin (Fig. 3d). This was due to three putative AA7 oxidoreductases all having a vanillyl-alcohol oxidase (VAO)-type FAD domain (#439950 20-fold, #446722 12-fold and #379025; no signal P ninefold) that were strongly induced in the presence of lignin (Additional file 2: Figure S3M) [48]. A respective 3.7- and 7.7-fold increase in two putative AA3 GMC oxidoreductases (#401428 and #420563; no signal P) further contributed to the overall increase in H_2_O_2_-producing enzymes (Additional file 2: Figure S3N). *P. anserina* has previously been shown to respond differently on various biomass sources regarding the induction of H_2_O_2_-producing enzymes [10, 16]. Our observations clearly show that lignin is amongst the inducing agents for these enzymes and this report constitutes the first experimental evidence of induction of AA7 oxidoreductases of *P. anserina*.

The characterized AA7 oxidoreductases have been listed in the CAZy database as FAD-linked oligosaccharide oxidases, and are structurally related to berberine-bridge enzyme (BBE)-like enzymes [15, 49]. However, we conducted a phylogenetic analysis of the VAO/PCMH superfamily according to Ewing et al. [50], which revealed that the secreted AA7 oxidoreductases of *P. anserina* markedly induced upon growth on lignin (#439950 and #446722) belong to other subclades of the family of BBE-like enzymes (Additional file 4: Figure S4). These subclades do not contain carbohydrate-active enzymes, but rather comprise specific alcohol oxidases that are involved in the biosynthesis of a diverse group of secondary metabolites that act as antibiotic or phytotoxin (See Additional file 4 for details). The reason for the upregulation of the *P. anserina* AA7 enzymes in the presence of lignin is not straightforward, but might be stress-related.

The presence of lignin caused a 20-fold overall increase in laccases, which was primarily due to a 63-fold induction of an AA1_3 laccase (#430065) (Additional file 2: Figure S3O). This enzyme has previously been annotated as a putative bilirubin oxidase, named *bod*1, but it was later found to actually correspond to a ‘new’ thermotolerant laccase after heterologous production and biochemical characterization [5, 51]. The biotechnologically interesting thermostability of the laccase was later further demonstrated by enzymatic assays following targeted mutations [52]. A mutant lacking the *bod*1 enzyme, furthermore, showed a mildly decreased ability to grow on lignin next to a decreased ability to resist various phenolics and H_2_O_2_ [52]. The strong induction of this enzyme in the presence of lignin observed here further underlines its potential involvement in the degradation of lignin. This laccase was also abundantly secreted when *P. anserina* was grown on other lignocellulosic substrates [10]. In fact, the enzyme was the most abundantly produced laccase when the fungus was grown on wheat straw. Note, however, that the enzyme was not included in the selected genes for analysis in the study of Mäkelä et al. [10].

Another laccase (#99820) was only produced in the presence of lignin, albeit in relatively low amount (Additional file 2: Figure S3O). The low abundance is in line with the results reported by Mäkelä et al., where only very minor amounts of this laccase could be detected when *P. anserina* was grown on soy bean hulls [10]. Despite the apparent low abundance, Xie et al. showed through targeted mutations that this enzyme is involved in the ability of the fungus to grow on wood shavings [17]. Note that, as already mentioned above, the (expected) relatively low redox potentials of these laccases consequently would have only allowed the enzymes to act on phenolic moieties in the absence of (natural) mediators.

Knowing that specific enzyme activities are not taken into account, the extensive induction of H_2_O_2_ producers at least suggests some involvement in the observed ligninolysis (Fig. 3). Although H_2_O_2_-producing enzymes have often been considered an accessory to peroxidases, the *P. anserina mat *+ genome encodes only peroxidases with low sequence homology to lignin, manganese and versatile peroxidases (LiP, MnP and VP) [5]. As such, a sole facilitating role of these H_2_O_2_ producers to enzymes involved in ligninolysis seems unlikely. However, the produced H_2_O_2_ might have been involved in LPMO action and, consequently, co-regulation between (some) H_2_O_2_ producing enzymes and LPMOs cannot be excluded [45, 53].

Alternatively, the produced H_2_O_2_ might have been involved in Fenton chemistry. The Fenton reaction describes the conversion of H_2_O_2_ to hydroxyl radicals in the presence of iron at acidic conditions. These highly reactive radicals are expected to result in ‘untargeted’ or ‘nonselective’ ligninolysis, a mechanism that is often associated with the action of brown-rot basidiomycetes [54, 55]. The fungal metabolite 2,5-dimethoxyhydroquinone has been shown to drive the Fenton reaction in the latter fungi and, accordingly, has been used as marker for the possible involvement of Fenton chemistry [54, 56, 57]. Though LC–MS analysis of the culture supernatants failed to detect this metabolite, other (lignin-derived) products might have fulfilled the same role in *P. anserina* biodegradation reactions. Although these secretome analyses cannot provide a definite answer on the underlying ligninolysis mechanisms, they do highlight potentially involved enzymes that are interesting candidates for follow-up gene-knockout and enzyme production/characterization studies.

## Conclusions

In summary, we have for the first time, provided unambiguous confirmation of the ligninolytic activity of the ascomycete fungus *Podospora anserina* through comprehensive substrate analyses of wheat straw lignin after growth of the fungus. Fungal growth resulted in substantial lignin removal and the accumulation of oxidatively degraded substructures. The insights obtained through substrate characterization agreed well with complementary proteomics analysis. Most importantly, the analysis showed that the secretome of *P. anserina* was considerably altered in the presence of lignin, with a strong reduction of cellulases and galactomannanases in particular, and suggested the potential involvement of laccases and H_2_O_2_ producing enzymes in ligninolysis reactions. Our work highlights several interesting candidate enzymes for further biochemical characterization and employment in biotechnological applications.

## Methods

### Materials

All chemicals and solvents were obtained from commercial suppliers and used as supplied. Water used in all experiments was purified via a Milli-Q water system (Millipore, Billerica, MA, USA).

### Preparation of wheat straw lignin isolate

Lignin was isolated from wheat straw by modifications of the classical lignin isolation procedures [58, 59]. Extractives were removed from milled wheat straw (< 1 mm) by sequential extraction with acetone and water. Extractive-free wheat straw (3 × 30 g) was planetary ball milled (PM100, Retsch, Haan, Germany) in a 500-mL zirconium dioxide jar containing 100 Φ 10 mm zirconium dioxide balls at a frequency of 600 rpm for a net milling time of 1 h in 10 min milling/20 min break interval cycles to prevent overheating of the material. Note that milling in the 500-mL jar setup proceeds with a higher intensity compared to routine 50-mL scale and, therefore, the same extent of milling is achieved in a much shorter time. The ball-milled material was subsequently water-extracted in a concentration of 5% (w/w) at 50 °C for 20 h under rotary shaking. The water-insoluble residue was obtained through centrifugation (18,000×*g*, 10 min, 20 °C) and washed two times with water before being suspended in 50 mM sodium acetate buffer pH 5 at a concentration of 5% (w/w). The suspension was incubated with the commercial enzyme cocktail ViscoStar 150 L (Dyadic, Jupiter, FL, USA) (0.125 mL/g biomass) at 50 °C for 24 h to degrade the cell wall polysaccharides. The insoluble material after incubation was obtained through centrifugation (18,000×*g*, 10 min, 20 °C), resuspended in buffer, loaded with fresh enzyme and incubated for another 24 h. After incubation, the suspension was centrifuged and the insoluble residue was washed three times with water before freeze-drying. The freeze-dried material was subsequently suspended in 80% (v/v) aqueous dioxane at 5% (w/w) dry matter loading and extracted at room temperature under nitrogen atmosphere for 24 h. The supernatant was recovered by centrifugation (30,000×*g*, 10 min, 20 °C) and the residue was extracted again. The supernatants were combined, freeze-dried, washed with water to remove traces of dioxane and freeze-dried again to yield the wheat straw lignin isolate.

### Growth of *Podospora anserina* on wheat straw lignin

Media and growth conditions for *P. anserina* were based on previously described procedures [21, 60]. Water-insoluble wheat glucuronoarabinoxylan (GAX, Megazyme, Wicklow, Ireland, 100 mg) and GAX (100 mg) combined with wheat straw lignin isolate (100 mg) were dispersed in 5 mL M2 minimal medium in 40-mL flasks and autoclaved prior to inoculation. *P. anserina* strain S mat + (CBS 143333) was grown on Luria–Bertani agar at 27 °C for 5 days before 0.5-cm plugs were taken from the formed mycelium to inoculate the samples. Flasks were statically incubated at 27 °C for 3 or 7 days. All experiments were performed in triplicate. After incubation mycelium was scooped out with a sterile loop and stored at − 20 °C. Culture samples (2.5 mL) were taken and centrifuged (10,000×*g*, 10 min, 4 °C) to separate the solid fraction from the supernatant for proteome analysis. Both fractions were stored at − 20 °C. The residual substrate in the flasks was removed by dispersion in water. Residual substrate and mycelium were water-extracted for 1 h at 4 °C under continuous stirring before centrifugation (60,000×*g*, 5 min, 20 °C) to separate water-soluble and insoluble fractions. The obtained fractions were flash-frozen in liquid nitrogen and freeze-dried.

### Quantitative py-GC–MS with ^13^C lignin as internal standard

Analytical pyrolysis coupled to gas chromatography with high-resolution mass spectrometric detection (Exactive Orbitrap, Thermo Scientific, Waltham, MA, USA) was performed as previously described [19, 61]. To each sample (~ 50 µg), 10 µL of a ^13^C wheat straw lignin internal standard (IS) solution (1 mg mL^−1^ ethanol/chloroform 50:50 v/v) was added and dried prior to analysis [22]. All samples were prepared and analyzed in duplicate. Lignin-derived pyrolysis products were monitored in full MS mode on the most abundant fragment per compound (both nonlabeled and uniformly ^13^C labeled). Pyrograms were processed by TraceFinder 4.0 software. Lignin contents and relative abundances of lignin-derived pyrolysis products were calculated as described previously [18, 19].

### HSQC NMR spectroscopy

For NMR analysis, biological triplicates were mixed in equal dry matter amounts to a single replicate. HSQC NMR spectra were recorded on a Bruker AVANCE III 600 MHz NMR spectrometer as previously described [19]. Spectra were processed according to del Río et al. [34].

### Carbohydrate content and composition

Constituent monosaccharides released after acid hydrolysis were determined in duplicate as previously reported by Englyst & Cummings with reported modifications [20, 62].

### Secretome extraction, protein preparation and analysis

For secretomic analysis, triplicate samples after 3 days of fungal growth were used. A 900-μL aliquot of water-soluble culture fraction was mixed with two volumes of methanol and proteins were left to precipitate overnight at − 20 °C. The precipitates were pelleted by centrifugation, washed by 60% (v/v) cold aqueous methanol, pelleted again and resuspended with 30 μL of anionic acid labile surfactant II (Protea Biosciences, Morgantown, WV, USA) in 200 mM ammonium bicarbonate. Twenty microliters of the resolubilized proteins were mixed with 5 μL 5X SDS-PAGE loading buffer (0.125 M Tris–HCl pH 6.8, 50% glycerol, 5% w/v SDS, 0.02% bromophenol and 0.35 M dithiothreitol), heated at 95 °C for 5 min before being loaded onto a 12% w/v acrylamide SDS-containing separating gel. Proteins were separated by SDS-PAGE until the 250-kDa marker of the BLUelf prestained protein ladder (FroggaBio, North York, Toronto, Canada) entered the separating gel. After the gel was stained with Coomassie brilliant blue, about 1 cm of the gel lane was cut out and polypeptides were in-gel digested with trypsin as described previously [63]. Peptide mixtures were resuspended in 45 μL 5% (v/v) aqueous acetonitrile with 0.1% (v/v) formic acid, containing 4 fmol/μL predigested bovine serum albumin. Five microliters of the peptide extract solution were subsequently injected onto a 75 μm × 15 cm C18 column and analyzed by LC–MS/MS by using an Easy-LC II Nano HPLC system in-line connected to a Velos LTQ-Orbitrap mass spectrometer (Thermo Fisher) as previously described [64]. Peptide and protein identification were performed by using Proteome Discoverer 2.2 software (Thermo Fisher) using the precursor ion quantification workflow with pairwise precursor ion peak area value-based protein abundance calculation. As additional criterion for protein abundance calculation, protein abundances were used only for those samples where at least one replicate had a minimum of two unique peptides identified. The relative abundance of the detected proteins was estimated by using the extracted precursor ion chromatogram peak areas of the identified peptides that were assigned to each protein. For relative abundance values to be considered significantly different, the requirements of a minimal twofold change and *p *< 0.05 from an unpaired *t*-test were used, as based on Daly et al. [64]. The *Podospora anserina* mat + v1.0 database from JGI was used. Functional annotations were assigned on the basis of InterPro descriptions (https://www.ebi.ac.uk/interpro/), Pfam predictions (https://pfam.xfam.org/) and Signal P prediction from JGI. CAZymes in the *P. anserina* mat + v1.0 genome were annotated on the basis of dbCAN (http://bcb.unl.edu/dbCAN2/) and Pfam predictions.

## Supplementary information

**Additional file 1:** Substrate characterization data: **Figure S1.** Py-GC-HR-MS pyrograms (TIC) of wheat straw, wheat straw lignin and glucuronoarabinoxylan (GAX). **Table S1.** Py-GC-HR-MS relative abundance of lignin compounds in wheat straw lignin isolate used for fermentations with *P. anserina*. **Table S2.** Semiquantitative ^1^H–^13^C HSQC NMR structural characterization of wheat straw lignin isolate used for fermentations with *P. anserina.*

**Additional file 2:** Proteomic analysis: **Figure S2.** Proteomic analysis of secreted proteins during growth of *P. anserina* on glucuronoarabinoxylan (GAX) and GAX + Lignin. Grouped according to functional annotations. **Figure S3.** Proteomic analysis of secreted individual proteins during growth of *P. anserina* on glucuronoarabinoxylan (GAX) and GAX + Lignin. Accession numbers according to JGI database (*P. anserina mat *+ v1.0).

**Additional file 3:** Proteomic analysis data. Raw and processed proteomic analysis data of secreted proteins during growth of *P. anserina* on glucuronoarabinoxylan (GAX) and GAX + Lignin. **Table S3.** Unique/induced proteins other than plant cell wall active CAZymes during growth of P. anserina on GAX or GAX + Lignin. Minimal relative abundance 0.2% of total protein and > twofold increase in absence/presence lignin.

**Additional file 4: Figure S4.** Phylogenetic analysis of lignin-induced *P. anserina* AA7 oxidoreductases.

## Data Availability

Supporting data will be provided as Additional files 1, 2, 3 and 4.

## Abbreviations

AA: Auxiliary activity
AGL: α-Galactosidase
AXE: Acetyl xylan esterase
BBE: Berberine-bridge enzyme
BGL: β-Glucosidase
CAZy: Carbohydrate-active enzymes
CBH: Cellobiohydrolase
CBM: Carbohydrate-binding module
CDH: Cellobiose dehydrogenase
CE: Carbohydrate esterase
^13^C-IS: Uniformly ^13^C-labeled internal standard
EGL: Endoglucanases
FADO: Flavin adenine dinucleotide linked oxidase
FAE: Feruloyl esterase
GAX: Glucuronoarabinoxylan
GH: Glycoside hydrolase
GLOX: Glyoxal oxidase
GMC: Glucose–methanol–choline
H_2_O_2_: Hydrogen peroxide
H_2_SO_4_: Sulfuric acid
HPLC: High-performance liquid chromatography
HSQC NMR: Heteronuclear single quantum coherence nuclear magnetic resonance
LPMO: Lytic polysaccharide monooxygenase
MAN: Endomannanase
MND: β-Mannosidase
py-GC–MS: Pyrolysis gas chromatography–mass spectrometry
SDS-PAGE: Sodium dodecyl sulfate-polyacrylamide gel electrophoresis
VAO: Vanillyl-alcohol oxidase
