# Supplementary material for: Evidence for ligninolytic activity of the ascomycete fungus Podospora anserina
Source: Biotechnol Biofuels. 2020 Apr 16;13:75. doi: 10.1186/s13068-020-01713-z (PMC7161253; doi:10.1186/s13068-020-01713-z)
Supplement: Supplementary file 1 — Additional file 1: Substrate characterization data: Figure S1. Py-GC-HR-MS pyrograms (TIC) of wheat straw, wheat straw lignin and glucuronoarabinoxylan (GAX). Table S1. Py-GC-HR-MS relative abundance of lignin compounds in wheat straw lignin isolate used for fermentations with P. anserina. Table S2. Semiquantitative 1H–13C HSQC NMR structural characterization of wheat straw lignin isolate used for fermentations with P. anserina. [file 13068_2020_1713_MOESM1_ESM.pdf]

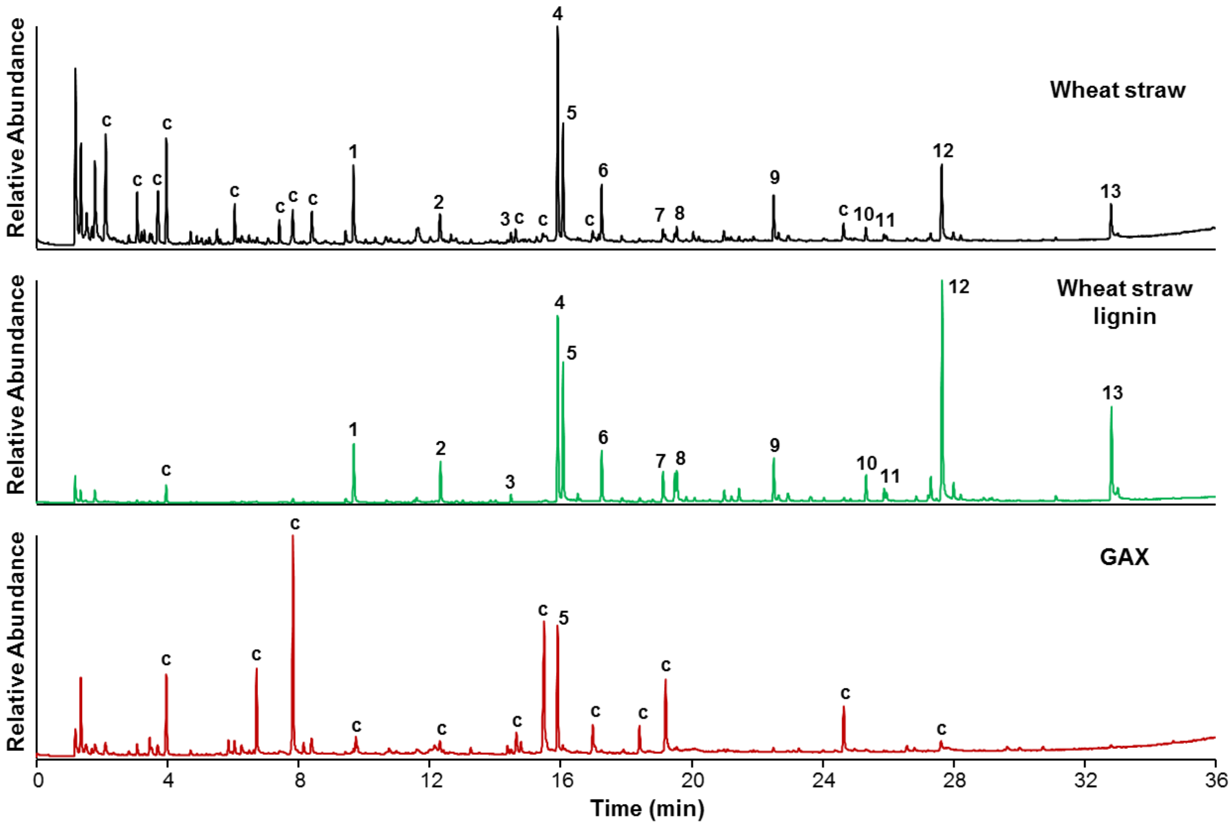

Figure S1. Py-GC-HR-MS pyrograms (TIC) of wheat straw, wheat straw lignin and glucuronoarabinoxylan (GAX). Numbers refer to major lignin-derived compounds: 1 guaiacol; 2 4-methylguaiacol; 3 4-ethylguaiacol; 4 4-vinylphenol; 5 4-vinylguaiacol; 6 syringol; 7 *trans*-isoeugenol; 8 vanillin; 9 4-vinylsyringol; 10 *trans*-propenylsyringol; 11 syringaldehyde; 12 *trans*-coniferyl alcohol; 13 *trans*-sinapyl alcohol. Carbohydrate-derived products (c) are not further specified.

Table S1. Py-GC-HR-MS relative abundance of lignin compounds in wheat straw lignin isolate used for fermentations with *P. anserina*. Corrected for relative response factors and relative abundance of ^{13}C internal standard analogues. Sum on the bases of structural classification according to van Erven et al. (1, 2). Average and standard deviation of analytical duplicates.

	**wheat straw lignin**
**Lignin subunits (%)**	
**H**	8.6 ± 0.3
**G**	58.9 ± 0.2
**S**	32.4 ± 0.2
**S/G**	0.55 ± 0.0
**Structural moieties (%)**	
**Unsubstituted**	5.6 ± 0.2
**Methyl**	3.4 ± 0.1
**Vinyl**	27.0 ± 0.5
**4-VP**[a]	7.1 ± 0.3
**4-VG**[b]	16.4 ± 0.1
**C_{α}-ox**	4.8 ± 0.0
**C_{β}-ox**[c]	1.1 ± 0.0
**C_{γ}-ox**	55.7 ± 0.8
**Miscellaneous**	3.5 ± 0.1
**PhC_{γ}**[d]	60.1 ± 0.7

[a] 4-vinylphenol. [b] 4-vinylguaiacol. [c] excluding diketones. [d] phenols with intact α,β,γ carbon side chain.

Table S2. Semiquantitative ^{1}H-^{13}C HSQC NMR structural characterization of wheat straw lignin isolate used for fermentations with *P. anserina*.

	**wheat straw lignin**
**Lignin subunits (%)**[a]	
**H**	3
**G**	63
**G_{ox}**	0
**S**	34
**S_{ox}**	0
**S/G**	0.5
**Hydroxycinnamates (%)**[b]	
***p*-coumarate**	8
**ferulate**	6
**Flavonolignin (%)**[b]	
**tricin**	15
**Lignin interunit linkages (%)**[b,c]	
**β-*O*-4' aryl ethers**	56 (91)
**β-5' phenylcoumarans**	4 (7)
**β-β' resinols**	1 (2)
**total**	62 (100)

[a] relative distribution of lignin subunits ($H+G+G_{ox}+S+S_{ox}=100$)
[b] relative volume integral of substructure versus volume integral of total lignin subunits
[c] relative distribution of total interunit linkages in parentheses

References

1. Van Erven G, Nayan N, Sonnenberg AS, Hendriks WH, Cone JW, Kabel MA. Mechanistic insight in the selective delignification of wheat straw by three white-rot fungal species through quantitative ^{13}C-IS py-GC–MS and whole cell wall HSQC NMR. Biotechnol Biofuels. 2018;11:262.
2. Van Erven G, Hilgers R, de Waard P, Gladbeek E-J, van Berkel WJH, Kabel MA. Elucidation of in situ ligninolysis mechanisms of the selective white-rot fungus *Ceriporiopsis subvermispora*. ACS Sustainable Chem Eng. 2019;7:16757-64.
