# Supplementary material for: Evidence for ligninolytic activity of the ascomycete fungus Podospora anserina
Source: Biotechnol Biofuels. 2020 Apr 16;13:75. doi: 10.1186/s13068-020-01713-z (PMC7161253; doi:10.1186/s13068-020-01713-z)
Supplement: Supplementary file 2 — Additional file 2: Proteomic analysis: Figure S2. Proteomic analysis of secreted proteins during growth of P. anserina on glucuronoarabinoxylan (GAX) and GAX + Lignin. Grouped according to functional annotations. Figure S3. Proteomic analysis of secreted individual proteins during growth of P. anserina on glucuronoarabinoxylan (GAX) and GAX + Lignin. Accession numbers according to JGI database (P. anserina mat + v1.0). [file 13068_2020_1713_MOESM2_ESM.pdf]

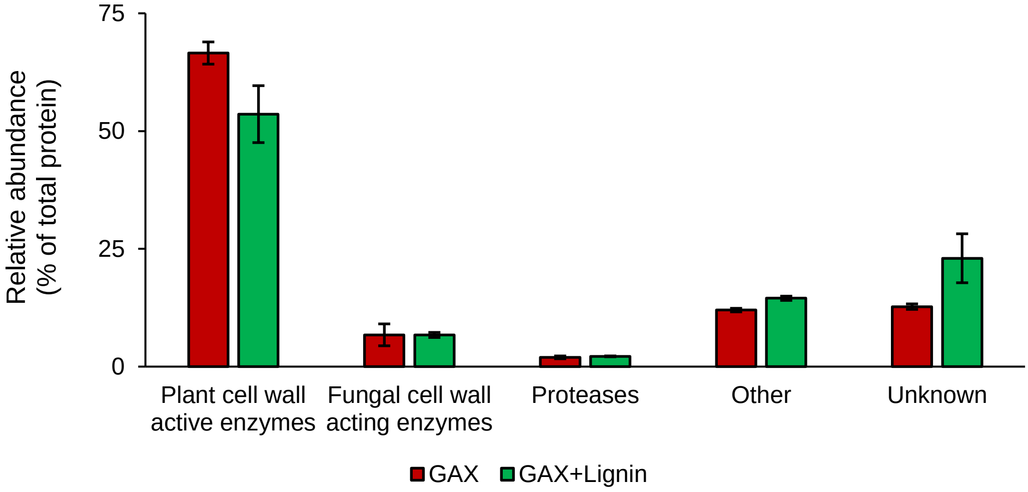

Figure S2. Proteomic analysis of secreted proteins during growth of *P. anserina* on glucuronoarabinoxylan (GAX) and GAX+Lignin**.** Grouped according to functional annotations in JGI database (*P. anserina mat*+ v1.0). Average and standard deviation of biological triplicates.

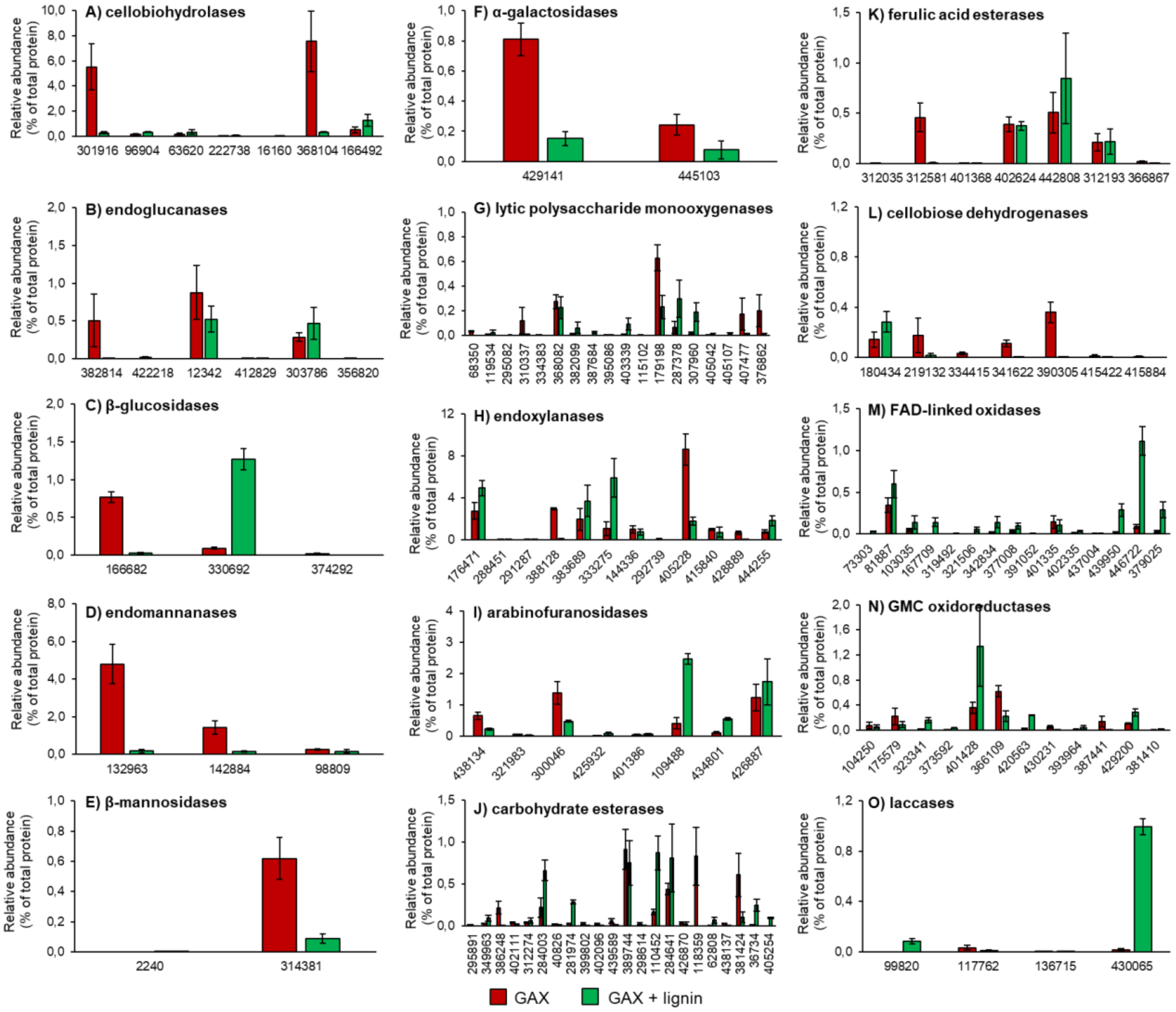

Figure S3. Proteomic analysis of secreted individual proteins during growth of *P. anserina* on glucuronoarabinoxylan (GAX) and GAX+Lignin**.** Accession numbers and functional annotations according to JGI database (*P. anserina mat*+ v1.0). Average and standard deviation of biological triplicates. Raw data in Additional File 3.
