# Supplementary material for: Evidence for ligninolytic activity of the ascomycete fungus Podospora anserina
Source: Biotechnol Biofuels. 2020 Apr 16;13:75. doi: 10.1186/s13068-020-01713-z (PMC7161253; doi:10.1186/s13068-020-01713-z)
Supplement: Supplementary file 4 — Additional file 4: Figure S4. Phylogenetic analysis of lignin-induced P. anserina AA7 oxidoreductases. [file 13068_2020_1713_MOESM4_ESM.pdf]

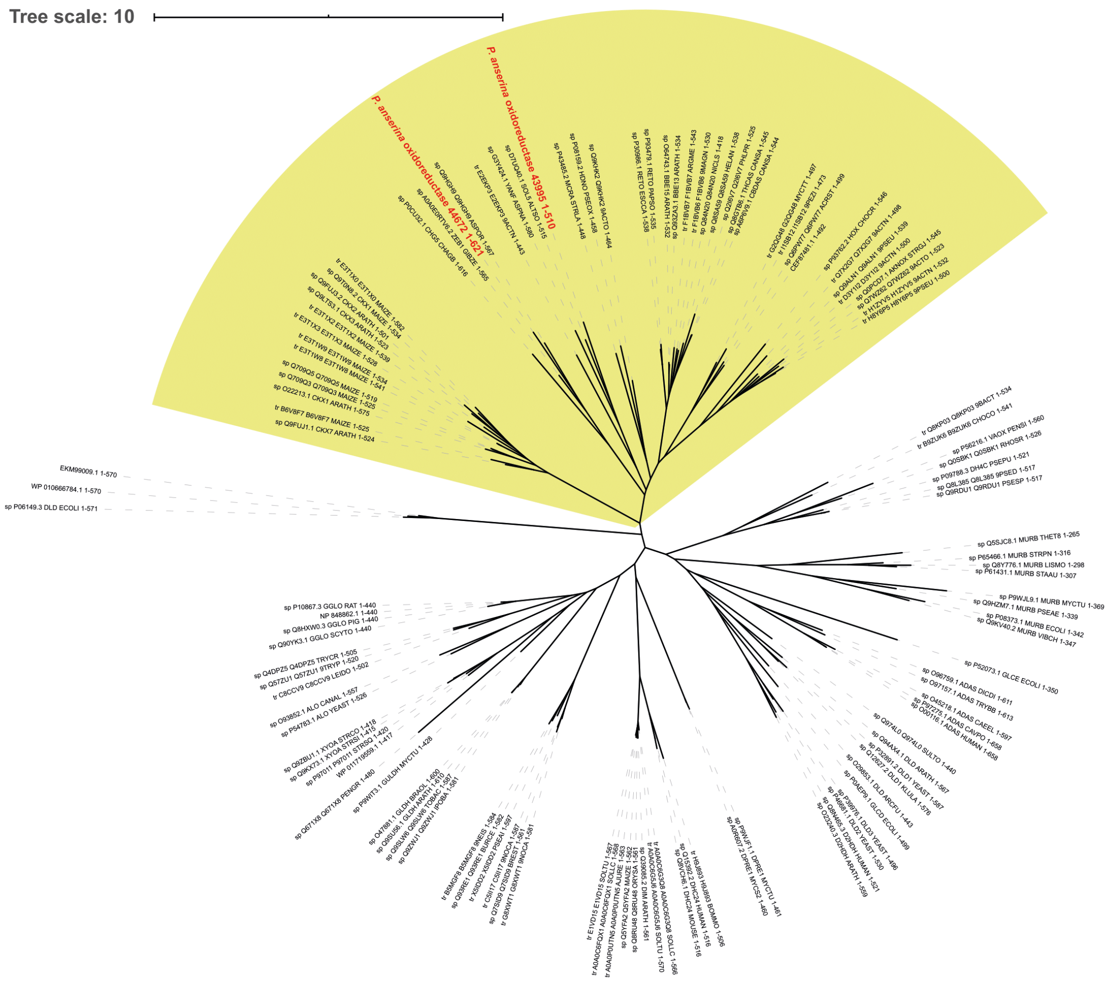

Figure S4. Phylogenetic analysis of lignin-induced *P. anserina* AA7 oxidoreductases (bold red). *P. anserina* enzymes presented as accession numbers according to JGI database (*P. anserina mat*+ v1.0), other enzymes according to UniProt (https://www.uniprot.org/) with enzymes belonging to the BBE-like family on yellow background. For details on the analysis of the VAO/PCMH superfamily, see Ewing et al. (1).

***P. anserina* AA7 oxidoreductase #44672 clusters with:**

- Name: FAD-linked oxidoreductase, UniProt: P0CU32 (CHG5 CHAGB), Gene: CHGG_01242-2, Organism: *Chaetomium globosum* ATCC 6205, Reference: (2)
- Name: FAD-linked oxidoreductase, UniProt: A0A0E0RTV6 (ZEB1_GIBZE), Gene: ZEB1, Organism: *Giberella zeae* PH-1, Reference: (3)
- Name: Isoamyl alcohol oxidase, UniProt: Q9HGH9 (Q9HGH9_ASPOZ), Gene: mreA, Organism: *Aspergillus oryzae*, Reference: (4)

***P. anserina* AA7 oxidoreductase #43995 clusters with:**

- Name: FAD-dependent oxidoreductase, UniProt: E2EKP3 (E2EKP3_STRC4), Gene: pac11, Organism: *Streptomyces coeruleorubidus*, Reference: n.a.
- Name: FAD-dependent monooxygenase, UniProt: G3Y424 (YANF_ASPNA), Gene: yanF, Organism: *Aspergillus niger* ATCC 1015, Reference: (5)
- Name: Bifunctional solanapyrone synthase, UniProt: D7UQ40 (SOL5_ALTSO), Gene: sol5, Organism: *Alternaria solani*, Reference: (6)

References

1. Ewing TA, Fraaije MW, Mattevi A, van Berkel WJ. The VAO/PCMH flavoprotein family. Arch Biochem Biophys. 2017;632:104-17.
2. Ishiuchi Ki, Nakazawa T, Yagishita F, Mino T, Noguchi H, Hotta K, et al. Combinatorial generation of complexity by redox enzymes in the chaetoglobosin A biosynthesis. J Am Chem Soc. 2013;135:7371-7.
3. Kim YT, Lee YR, Jin J, Han KH, Kim H, Kim JC, et al. Two different polyketide synthase genes are required for synthesis of zearalenone in *Gibberella zeae*. Mol Microbiol. 2005;58:1102-13.
4. Yamashita N, Motoyoshi T, Nishimura A. Molecular cloning of the isoamyl alcohol oxidase-encoding gene (mreA) from *Aspergillus oryzae*. J Biosci Bioeng. 2000;89:522-7.
5. Holm DK, Petersen LM, Klitgaard A, Knudsen PB, Jarczynska ZD, Nielsen KF, et al. Molecular and chemical characterization of the biosynthesis of the 6-MSA-derived meroterpenoid yanuthone D in *Aspergillus niger*. Chem Biol. 2014;21:519-29.
6. Kasahara K, Miyamoto T, Fujimoto T, Oguri H, Tokiwano T, Oikawa H, et al. Solanapyrone synthase, a possible Diels–Alderase and iterative type I polyketide synthase encoded in a biosynthetic gene cluster from *Alternaria solani*. ChemBioChem. 2010(9):1245-52.
